# Factors Associated with Food Insecurity in Households of Public School Students of Salvador City, Bahia, Brazil

**Published:** 2013-12

**Authors:** Liliane de Souza Bittencourt, Sandra Maria Chaves dos Santos, Elizabete de Jesus Pinto, Marie Agnès Aliaga, Rita de Cássia Ribeiro-Silva

**Affiliations:** Escola de Nutrição da Universidade Federal da Bahia. Av. Araújo Pinho 32, Canela. CEP 40.110-150 Salvador, Bahia, Brazil

**Keywords:** Food insecurity, Socioeconomic inequalities, Students, Brazil

## Abstract

This cross-sectional study was conducted to find out the factors associated with food insecurity (FI) in households of the students aged 6-12 years in public schools of Salvador city, Bahia, Brazil. The study included 1,101 households. Food and nutritional insecurity was measured using the Brazilian Food Insecurity Scale (BFIS). Data on socioeconomic and demographic characteristics as well as environmental and housing conditions were collected during the interviews conducted with the reference persons. Multivariate polytomous logistic regression was used in assessing factors associated with food insecurity. We detected prevalence of food insecurity in 71.3% of the households. Severe and moderate forms of FI were diagnosed in 37.1% of the households and were associated with: (i) female gender of the reference person in the households (OR 2.21, 95% CI 1.47-3.31); (ii) a monthly per-capita income below one-fourth of the minimum wage (US$ 191,73) (OR 2.63, 95% CI 1.68-4.08); (iii) number of residents per bedroom below 3 persons (OR 1.91, 95% CI 1.23-2.96); and (iv) inadequate housing conditions (OR 1.84, 95% CI 1.12-4.49). Socioeconomic inequalities determine the factors associated with FI of households in Salvador, Bahia. Identifying vulnerabilities is necessary to support public policies in reducing food insecurity in the country. The results of the present study may be used in re-evaluating strategies that may limit the inequalities in school environment.

## INTRODUCTION

In accordance with the law of Brazil (LOSAN-11.346/06), Food and Nutrition Security (FNS) is defined as the realization of the right to quality food in sufficient quantity without compromising access to other essential needs, such as health-promoting food practices that respect cultural diversity and are environmentally, economically and socially sustainable (1). The worldwide food crisis and economic recession during 2006-2008 hindered the goal of reducing the number of hungry people throughout the world. Therefore, it is not surprising to know that a total of 850 million people were facing food restriction with more than 98% living in developing regions as reported by the United Nations Food and Agriculture Organization (FAO) (2). Latin America accounts for approximately 38 million of the total number of hungry people worldwide, despite a reduction of 17.2% in this number between 1990 and 2008 (2). In this region, Brazil had the greatest reduction in food insecurity (31.5%) during that period. Nonetheless, data from the National Household Sample Survey (Pesquisa Nacional por Amostra Domiciliar-PNAD) conducted by the Brazilian Institute of Geography and Statistics (Instituto Brasileiro de Geografia e Estatística-IBGE) in 2009, using the Brazilian Food Insecurity Scale (Escala Brasileira de Insegurança Alimentar-EBIA) (3) showed that 30.2% of Brazilian private households were in a state of food insecurity (FI), with 5% of them in a situation of severe FI. In the state of Bahia, out of the total number of households with FI (47.4%), 9.7% showed severe FI (4). Food insecurity is associated not only with non-availability of food but also with social vulnerability, which includes a combination of socioeconomic and demographic variables (5). As a result, this causes a reduction in the level of familial well-being and, thus, negatively impacts the quality of life (6-9), making it impossible for a considerable portion of the population to have the financial resources to meet their basic nutritional needs. A number of studies have examined the impact of FI on health conditions, especially among children and adolescents. These studies indicate that FI is associated with an impairment of dietary patterns (10), nutritional status (11-14), physical and mental development (8,13), an increase in susceptibility to infectious diseases (15), and the risk of chronic diseases in adulthood (6,16). In addition to child labour, begging, and domestic violence, the FI may result in increasing the rates of failure and dropout in schools (17,18). Brazil has been implementing the National School Food Programme (Programa Nacional de Alimentação Escolar-PNAE) for more than 50 years. The programme aims at complementing pupil's diet to contribute to a better performance at school and correct potential food deficiencies at home. Thus, the PNAE is a programme that is relevant to Food and Nutrition Security. In the National Study on Food Security (4), households with at least one member below the age of 18 years, i.e. the target population of the PNAE, showed to have food insecurity prevalence higher than that in households with adults only (4). This result was observed in all regions of Brazil, with a higher risk of food insecurity among households in the northeastern region. Furthermore, considering the fact that the reduction in food availability at home tends to compromise the quality and quantity of food for this age-group (19), this study focused on household food insecurity for school children. Therefore, it is expected that, by identifying the determinants of FI in the households of school children, new PNAE guidelines can be developed and be aimed at promoting health and healthy food habit.

## MATERIALS AND METHODS

### Study design

This cross-sectional study was performed with families who have students between the ages of 7 and 14 years of either sex. These students were identified from a larger study that aimed at examining the factors associated with iron-deficiency anaemia in children and adolescents enrolled in public schools of Salvador city (20).

### Sample-size and sampling procedure

The sampling procedure in the original study involved a complex design, including the stratification of schools into two levels (state and municipal), which was followed by three stages of clustering the health districts, schools, and students. With the field-level logistics support, students were selected in 6 out of 12 districts of Salvador where 117 state schools and 173 municipal schools were identified. The number of students in state schools was 58,059 while the number of students in municipal schools was 56,555. To estimate the sample, we used data from the 2007 school census available through Bahia Education Secretary. A total of 10 students were selected from each of the 58 municipal schools, and 23 were selected from each of the 27 state schools to meet the previously-defined sample-size of 1,201 students. However, out of the total number of students initially selected, study could not be completed on 100 (8.3%) students. The reasons for this attrition resulted from refusal of students to participate in the study, relocation to another city/community or transfer to a different school. Thus, the final sample-size was 1,101.

The original study used for the sample data did not aim at evaluating the factors associated with food insecurity of students’ households. Therefore, for the present study, it was necessary to calculate the sample-size power in order to detect the associations between food insecurity and socioeconomic conditions of the households. The calculated power was of 99% (1-β), and the significance level was of 0.05 (1-α).

Data were collected between August and December 2007 by qualified and previously-trained personnel. The managers of the selected schools received an invitation letter to participate in the study. The letter contained objectives and research methodology of the study. Additional meetings for clarification were held, and consents were obtained both from schools and parents. The parents were also invited to attend the interviews at school.

### Training of interviewers

The interviewers of the research team were trained on data collection. After they were trained, a pilot study was conducted for field logistics adjustment after checking the measurement techniques and instruments. Pilot study participants were not included in the final sample of the current study. Throughout the field work, supervisors periodically assessed the performance of the interviewers to minimize possible errors in data collection.

### Dependent variable

Food and nutrition insecurity was measured using the Brazilian Food Insecurity Scale (BFIS). This scale has been adapted and validated for the Brazilian non-institutionalized population (3)(21). Questions concerning food insecurity were answered by the person responsible for feeding the family. The BFIS is an additive scale, and results were obtained by adding all scores on 15 items. The scale measures the extent to which the household is experiencing food insecurity, ranging from running out of food to lowering the quality of diet, and, ultimately, consuming very little or no food because of economic limitations. Responses to these items are also used in assessing different degrees of food insecurity (mild, moderate, and severe) experienced by families. We considered the presence or absence of residents below 18 years of age in the households when determining the level of food insecurity based on the scale score (22). In the households with residents below 18 years of age, the following cutoff points were used: 1-5 (mild FI), 6-10 (moderate FI), and 11-15 (severe FI). A BFIS score of zero indicated food-secure. These cutoff points were proposed by Radimer *et al*. (23) on the basis of an algorithm, validated for use within Brazil by researchers and applied for the Food Security National Survey.

### Independent variables

Data on socioeconomic and demographic characteristics as well as environmental and housing conditions were collected during the interviews conducted with the adults responsible for the students. The responsible adults were invited to come to the school for interview. The interviews were performed by trained and qualified interviewers who recorded the responses in a standardized questionnaire.

Socioeconomic and demographic characteristics included environmental index as well as the number of people living in the household (<4 reference category, 4-6, ≥7 residents), the number of residents below 15 years of age (≥4, <4 reference category), the number of residents per bedroom (≥3, <3 reference category), the per-capita monthly income in number of minimum wage (MW) (<1/4 MW, ≥1/4 MW reference category), and household reference member's (HRM's) educational level (≤4th grade, 5th to 8th grade, college or university level reference category). The study also included variables linked to the gender of the HRM (male reference category, female), and to his/her race or skin colour (white reference category, black, mixed or other). Race or skin colour, as defined by the Brazilian Institute of Geography and Statistics (IBGE), is determined upon the declaration of the interviewed person (4).

Data on home (ownership status of the house, type of construction, main floor-material, main materials in the roof and part of the house, number of residents per room), and basic sanitation characteristics (water supply, garbage collection and sanitary sewage system) were collected to prepare the environmental index, which was adapted from the model proposed by Issler and Giugliani (24). A score was attributed to each situation—the most favourable situations received 0, and the most unfavourable situations received 1. The sum of these values determined the index of environmental and housing conditions. This index, ranking from 0 to 16, was divided into two categories where ≤4 was considered adequate, and >4 was considered inadequate. According to this method, the housing conditions of 41.2% of the surveyed households were considered inappropriate.

### Statistical analysis

The analysis was performed using two approaches: descriptive analysis and regression modelling to determine the factors associated with food insecurity. First, we conducted polytomous logistic regression, with a dependent variable being FI, categorized as ‘food security’, 'mild food insecurity’ and 'moderate/severe food insecurity’. We provide interpretations of the odds ratios with 95% confidence interval derived from the polytomous regression model, using food insecurity as the reference category. Predictor variable conceptuals were first selected on the basis of national research results and models for food insecurity. Second, for entering predictor variables in the multivariate multinomial logistic model, a significance level of 20% was used (25). The variables were adjusted to the model by the backward stepwise method in the multivariate multinomial logistic regression, using a significance level of 5%. Statistical analyses were corrected by the complex sample design, using the set of SVY (Stata Survey) commands in STATA software (version 9.0).

### Ethical issues

The study protocol was approved by the Universidade Federal da Bahia Institute of Collective Health Research Ethics Committee. Parents or responsible adults who agreed to participate in the study signed an informed consent form (CEP-ISC/043-05).

## RESULTS

This study included 1,101 households with children aged between 7 and 14 years studying in public schools. According to the results of BFIS, it was found that 71.3% of this population was experiencing food insecurity. This percentage is divided into two levels of severity—34.2% had mild and 37.1% had moderate to severe level of food insecurity. Descriptive characteristics of the study participants are shown in Table 1.

The results of the polytomous logistic regression analysis are presented in Table 2. There was evidence of a positive association between mild FI and (i) female gender (OR 1.69, 95% CI 1.18-2.39), and (ii) skin colour of the HRM being mixed/others (OR 2.05, 95% CI 1.04-4.02). There was also a positive association between moderate/severe FI and (i) the HRM being female (OR 2.90, 95% CI 2.04-4.10), (ii) the HRM having less than 4 years of schooling (OR 2.55, 95% CI 1.64-3.94), (iii) skin colour of the HRM being black (OR 2.67, 95% CI 1.28-5.58), (iv) skin colour of HRM being mixed/other (OR 2.25, 95% CI 1.12-4.49), (v) a monthly per-capita income of less than 1/4 of the minimum wage (OR 4.69, 95% CI 3.25-6.77), (vi) households with 5 or 6 members (OR 2.10, 95% CI 1.43-3.08), (vii) households with more than 7 members (OR 2.40, 95% CI 1.50-3.82), (viii) households with more than 4 residents below 15 years of age (OR 2.17, 95% CI 1.38-3.37), (ix) households with more than 3 residents per room (OR 3.17, 95% CI 2.19-4.57), and (x) an indicator of inadequate housing conditions (OR 2.42, 95% CI 1.69-3.41).

**Table 1.** Characteristics of the households with students in public schools of Salvador-BA (2007)

Variable	n	%
Gender of the household reference member* (a)		
Male	580	52.7
Female	520	47.3
Education of the household reference member* (b)		
College or university level	300	28.7
5th to 8th grade	360	35.1
≤4th grade	378	36.2
Skin colour of the household reference member* (c)		
White	67	7.0
Black	275	28.7
Mixed and other	615	64.3
Per-capita monthly income* (d)		
>¼ MW	647	59.0
≤¼ MW	449	41.0
Number of residents in the household (e)		
≤4	516	46.9
5-6	381	34.6
≥7	203	18.5
Number of residents below 15 years of age* (f)		
<4	888	81.4
≥4	203	18.6
Number of residents per bedroom		
<3	714	64.9
≥3	387	35.1
Indicator of housing conditions* (g)		
Suitable	647	58.8
Inappropriate	453	41.2

*Missing data: a=1, b=57, c=144, d=342, f=10, g=1; MW=Minimum wage (US$ 191,73)

**Table 2.** Odds ratios of the association between food insecurity, socioeconomic, demographic, housing conditions and sanitation of the households with students in public schools of Salvador-BA (2007)

Variable	Mild FI*			Moderate/severe FI*		
	OR	95% CI	p value	OR	95% CI	p value
Gender of the household reference member						
Male	1			1		
Female	1.69	1.18-2.39	0.004	2.90	2.04-4.10	<0.001
Education of the household reference member						
College or university level	1			1		
5th to 8th grade	1.24	0.82-1.87	0.306	1.50	0.72-2.32	0.069
≤4th grade	1.14	0.73-1.77	0.553	2.55	1.64-3.94	<0.001
Skin colour of household reference member						
White	1			1		
Black	1.87	0.90-3.88	0.91	2.67	1.28-5.58	0.009
Mixed and other	2.05	1.04-4.02	0.036	2.25	1.12-4.49	0.022
Per-capita monthly income						
>¼ MW	1			1		
≤¼ MW	1.25	0.85-1.82	0.255	4.69	3.25-6.77	<0.001
Number of residents in the household						
≤4	1			1		
5-6	1.35	0.91-1.97	0.127	2.10	1.43-3.08	<0.001
≥7	0.86	0.52-1.42	0.557	2.40	1.50-3.82	<0.001
Number of residents below 15 years of age						
<4	1			1		
≥4	1.06	0.64-1.72	0.828	2.17	1.38-3.37	0.001
Number of residents per bedroom						
<3	1			1		
≥3	1.24	0.83-1.82	0.284	3.17	2.19-4.57	<0.001
Indicator of housing conditions						
Suitable	1			1		
Inappropriate	1.20	0.83-1.72	0.321	2.42	1.69-3.41	<0.001

*Comparison group: Food security situation; MW=Minimum wage; OR=Odds ratio

In the final multivariate model, the moderate/severe FI remained associated with female gender of the HRM (OR 2.21, 95% CI 1.47-3.31), a monthly per-capita income below 1/4 of the minimum wage (OR 2.63, 95% CI 1.68-4.08), households with more than 3 residents per bedroom (OR 1.91, 95% CI 1.23-2.96), and an indicator of inadequate housing conditions (OR 1.84, 95% CI 1.12-4.49). Lower educational levels of HRM were also associated with moderate/severe FI but with a borderline level of statistical significance (OR 1.68, 95% CI 1.00-2.81). The mild FI was associated with female gender (OR 1.60, 95% CI 1.09-2.34), and skin colour of the HRM being mixed/others (OR 2.08, 95% CI 1.01-4.25) (Table 3). The goodness of fit test indicated that the model showed satisfactory calibration (p>0.05).

## DISCUSSION

The prevalence of food insecurity found among the families of students in Salvador (71.3%) was higher than the prevalence found in other regions covered in the PNAD 2004/2009 for Brazil (30.2%) (4). The prevalence of food insecurity was 46.1% in the northeast, 40.3% in the north, 30.1% in the midwest, 23.3% in the southeast, and 18.7% in the south (4). In addition, studies in the Forest Zone of Pernambuco found the prevalence of food insecurity in Gameleira and São João do Tigre to be 88.2% (26) and 87.3% (27) respectively. Conversely, a lower prevalence of food insecurity was found in the study conducted in Duque de Caxias in the metropolitan region of Rio de Janeiro (53.8%) (28).

The aim of this study was to explore the factors associated with FI. The results of the multivariate analysis of FI showed an association with gender of the reference person in the households, educational level and the skin colour of the HRM as well as monthly per-capita income, the number of residents per bedroom, and an indicator of housing conditions.

**Table 3.** Adjusted odds ratios to assess the factors associated with food insecurity of households with students in public schools of Salvador-BA (2007)

Variable	Mild FI*		Moderate/severe FI*	
	AOR	95% CI	AOR	95% CI
Gender of the household reference member				
Male	1		1	
Female	1.60	1.09-2.34	2.21	1.47-3.31
Education of the household reference member				
College or university level	1		1	
5th to 8th grade	1.12	0.71-1.75	1.27	0.76-2.09
≤4th grade	0.79	0.60-1.57	1.68	1.00-2.81
Skin colour of household reference member				
White	1		1	
Black	1.81	0.84-3.92	1.92	0.88-4.17
Mixed/other	2.08	1.01-4.25	1.78	0.85-3.70
Per-capita monthly income				
>¼ MW	1		1	
≤¼ MW	1.07	0.67-1.69	2.63	1.68-4.08
Number of residents per bedroom				
<3	1		1	
≥3	1.06	0.66-1.67	1.91	1.23-2.96
Indicator of housing conditions				
Suitable	1		1	
Inappropriate	1.22	0.86-1.83	1.84	1.12-2.08

*Comparison group: Food security situation; AOR=Adjusted odds ratio; MW=Minimum wage

If the household reference person was female, the household was 1.60 times (95% CI 1.09-2.34) more likely to be classified in the mild food insecurity category and 2.21 times (95% CI 1.47-3.31) more likely to be classified in the moderate and severe food insecurity category. These results are consistent with those identified in other studies conducted in Brazil (9), Colombia (5), and Bangladesh (29), in which the families with female heads were more vulnerable to food insecurity. The association between female gender of the household head and food insecurity has been addressed in debates about poverty and gender (30). In studies on poverty, women as heads of the households are used as a measure of feminization of poverty (30). Some reasons for this association are attributed especially to the lower income earned by women in their workplace (30).

If the HRM only had up to 4th grade of education, the household was 1.68 times (95% CI 1.00-2.81) more likely to have moderate to severe food insecurity. Similar results regarding determinants of moderate and severe food insecurity were observed in studies performed in Campinas, São Paulo (31), and Duque de Caxias, Rio de Janeiro (28). The negative influence of limited education on food insecurity was also observed in other studies conducted in regions of Texas, USA (32) as well as in other countries (33,34). In addition to higher education, better employment in terms of employment rate, salary levels, and employment stability can contribute to more stable financial resources and, thus, enable families to have a better access to food. In addition, higher educational levels may enhance cognitive abilities, which may result in an increased access to information that may improve the quality of life for all family members.

If the skin colour of the HRM was mixed/other, the household was 2.08 (95% CI 1.02-4.25) times more likely to have mild food insecurity. In addition, there was a statistically-significant association between black and mixed/other skin colour of the HRM and moderate/severe food insecurity (9). This association was observed throughout all units of the federation, especially in the north and northeast of the country. Similar results were observed in the Campinas study (31), reinforcing the evidence that people with black and mixed skin colour in Brazil are the most vulnerable in terms of social and economic conditions (35).

Households of students enrolled in public schools in Salvador that earned less than 1/4 of the minimum wage were 2.63 times (95% CI 1.68-4.08) more likely to be in a moderate to severe food insecurity situation. These results are comparable to data presented by Marin-Leon *et al*. (9) for the country, which revealed that, in most households where the income did not exceed this value, moderate or severe food insecurity was experienced. Within this low per-capita monthly income category, moderate or severe food insecurity reached 59.5% of the households while, among those having a monthly per-capita income above four times the minimum wage, only 1% experienced food insecurity. Similar results were reported by several authors who have addressed this issue (32,36).

In this study, households with three or more persons per bedroom were 1.91 times more likely to experience moderate or severe food insecurity than households that had less than three people per bedroom. A similar result was found in the study performed in Campinas (18) where the likelihood of moderate or severe food insecurity was 5.2 times greater for each additional person per bedroom. This demonstrates that crowding in the household contributes to food insecurity. In agreement with this study, the FI status was associated directly with the number of family members in other studies (33). The association between these variables can be clearly observed in special situations, such as high food prices or temporary unemployment. These factors result in a decrease in the food supply at home. Therefore, in larger families, there will be less food available for each member (33).

In this study, the indicator of housing conditions, which is based on housing characteristics and sanitation conditions, revealed that an inadequate environment leads to 1.84 times higher chance (1.12 to 2.08) of being classified in the moderate or severe food insecurity category. In studies that examined housing conditions individually in relation to food insecurity, there was association between housing conditions and food insecurity (37).

### Limitations

The limitations of this study were primarily due to study design. Indeed, we emphasize that this cross-sectional study cannot establish a causal relationship due to the temporal sequence between exposure and effect. Another possibility would have been to limit the scale used in evaluating the FI. However, the scale is useful to estimate the prevalence of various levels of food insecurity, to identify risk groups or populations at local, regional or national levels and to study the determinants and consequences of food insecurity and so can be used for policy-making (38,39).

### Conclusions

The results showed a high prevalence of food insecurity among households with school children. This result was associated with characteristics of the household reference member and with the living conditions at home. In general, the distribution of food insecurity is similar to the distribution pattern of inequality and, therefore, similar variables that result in limited access to healthcare and services also limit access to food resources. The results of the present study may be used in re-evaluating strategies that may limit the inequalities in the social environment of schools.

## ACKNOWLEDGEMENTS

The study was funded by Conselho Nacional de Desenvolvimento Científico e Tecnológico (CNPq) [processo n. 402462/2005-0]
